# What Italian Defense Attorneys Know about Factors Affecting Eyewitness Accuracy: A Comparison with U.S. and Norwegian Samples

**DOI:** 10.3389/fpsyt.2013.00028

**Published:** 2013-05-16

**Authors:** Svein Magnussen, Martin A. Safer, Giuseppe Sartori, Richard A. Wise

**Affiliations:** ^1^Cognitive Developmental Research Unit, Department of Psychology, University of OsloOslo, Norway; ^2^Department of Psychology, The Catholic University of AmericaWashington, DC, USA; ^3^Department of Psychology, University of PaduaPadua, Italy; ^4^Department of Psychology, University of North DakotaGrand Forks, ND, USA

**Keywords:** eyewitnesses, memory, professional beliefs, attorneys

## Abstract

We surveyed 100 Italian defense attorneys about their knowledge and beliefs about factors affecting eyewitness accuracy. The results of similar surveys show that U.S. defense attorneys were significantly more knowledgeable than other legal professionals, including U.S. prosecutors and U.S. and European judges. The present survey of Italian defense attorneys produced similar results. However, the results suggest that the defense attorney’s superior performance may be due at least in part to their skepticism of eyewitness testimony rather than their greater knowledge of eyewitness factors.

## Introduction

There is a growing concern among eyewitness researchers that legal professionals lack sufficient knowledge about memory and the many factors that may affect eyewitness accuracy to prevent wrongful convictions from eyewitness error (Benton et al., 2007; Wise et al., 2007). The list of historical and contemporary examples of persons wrongfully convicted by eyewitness testimony is long indeed. For example, in 1796 Joseph Lesurques, a retired army officer, was wrongfully executed for the robbery and brutal murders of mail coach attendants. Two eyewitnesses who erroneously identified him as one of the bandits while he was in the waiting room of the magistrate who was hearing the case caused his wrongful conviction (Sporer et al., 1996). Another famous example of eyewitness error occurred 100 years later, in 1896, when a London court convicted Adolf Beck, a Norwegian citizen, for swindling women out of their jewelry after 12 of the victims erroneously identified him in a lineup. The Beck case led to major reforms including the creation of the Court of Criminal Appeal. Many judges in Commonwealth countries still cite the case as a glaring example of the dangers of eyewitness testimony (Coates, 2001).

Such cases may be well known historical examples of eyewitness error, but eyewitness errors happen with alarming frequency in current criminal cases. For example, eyewitness error occurred in more than 75% of the 302 DNA exoneration cases in the U.S. (Saks and Koehler, 2005)^1^. Moreover, studies of wrongful felony convictions in the U.S. prior to the DNA exoneration cases show that eyewitness error is involved in half or more of all wrongful felony convictions (Borchard, 1932; Huff, 1987; Rattner, 1988).

Concern about the ability of criminal justice systems to mitigate eyewitness errors has been exacerbated by recent surveys in China, Estonia, Norway, Sweden, and U.S. of judges, law enforcement officers, and attorneys (Wise and Safer, 2004; Granhag et al., 2005; Benton et al., 2006; Magnussen et al., 2008; Wise et al., 2009, 2010, 2011; Kask, 2011). These surveys show that in general legal professionals’ knowledge of eyewitness testimony does not exceed the knowledge of students, jury members, or prospective jury members (Benton et al., 2006; Magnussen et al., 2010; Wise and Safer, 2010; see also Desmarais and Read, 2011). It also differs significantly from the knowledge of eyewitness experts (Kassin et al., 2001).

Moreover, sizable minorities or even majorities of the legal professionals disagree with eyewitness experts even when there is a strong consensus among experts about how an eyewitness factor affects accuracy, and there is strong empirical support for their consensus. It is the frequency of legal professionals’ wrong answers rather than their “do not know” responses that is especially alarming. These results suggest that many legal professionals rely on common sense and intuition when evaluating eyewitness accuracy rather than scientific research.

The single exception to the general pattern of results for legal professionals’ knowledge about eyewitness testimony is U.S. defense attorneys. They performed significantly better than U.S. prosecutors and the other legal professions (Wise et al., 2009) and closer to the eyewitness experts (Kassin et al., 2001). Does the superior performance of the U.S. defense attorneys reflect greater knowledge of eyewitness factors, or does it simply indicate a more skeptical attitude about the accuracy of eyewitness testimony? After all, it is defense attorneys’ responsibility to challenge the validity of the state’s evidence, and therefore they are likely to be more skeptical than other legal professionals of the accuracy of eyewitness testimony. Wise et al. (2009) tested the latter hypothesis by distributing the questionnaire to a sample of undergraduate students who were instructed to take a skeptical attitude toward eyewitnesses. They found some support for the hypothesis that defense attorneys’ superior performance was partially a product of skepticism, rather than greater knowledge.

Still, the superior performance of the U.S. defense attorneys might be a chance finding, or it may only apply to U.S. defense attorneys. For instance, the DNA exonerations cases in the U.S. may have sensitized U.S. defense attorneys to the problem of eyewitness error. We conducted a study of Italian defense attorneys, using a slightly modified version of the Wise and Safer questionnaire (Wise and Safer, 2004; Magnussen et al., 2008, 2010) translated into Italian. Italian defense lawyers tend to specialize in criminal law and accept mainly criminal cases. If the superior knowledge of defense attorneys is associated with their professional role, we would expect the beliefs and knowledge of the Italian defense attorneys to be similar to the U.S. defense attorneys and the eyewitness experts rather than the other U.S. and European legal professionals.

### The Italian and U.S. criminal justice systems

The Italian criminal justice system is a mixture of the inquisitorial and adversarial systems of justice. For example, though Italian prosecutors are responsible for collecting evidence, judges can also take an active role in gathering evidence and can use their own expert to provide advice on scientific or technical evidence. In Italy, the criminal process is divided into two phases: the investigative phase and the trial phase. The preliminary investigation begins either when a crime is reported to the public prosecutor, or the prosecutor or law enforcement agency discovers sufficient evidence of a crime. The preliminary investigative phase has a maximum time limit, usually 6 months, which begins to run when the name of the suspect is officially recorded. The judge for preliminary investigations supervises the pre-trial phase of a criminal trial in Italy and determines if there is sufficient evidence to take to a case to trial.

The Italian judiciary is independent and once appointed judges serve for life. Italy has a three tier court system that consists of trial courts, intermediate appellate courts, and a supreme court. In Italy, citizens, called lay judges, are involved in the determination of a defendant’s guilt in only the most serious criminal cases, where the maximum sentence is more than 24 years. In these cases, 2 professional judges and 11 citizens randomly selected render the verdict in the case. Their verdict does not have to be unanimous. In all other criminal cases, either one judge or three professional judges decide the case depending on the seriousness of the crime. Lay judges also help decide appeals in the Court of Assizes of Appeals but not in Italy’s other intermediate appellate courts. The highest Italian court is the Court of Cassation, and it can only review lower court decisions on the law not the facts.

The U.S. has an adversarial system of justice. The parties to a criminal action are primarily responsible for gathering and presenting the evidence in a criminal case. Although judges can call witnesses and ask questions of witnesses, they rarely do so especially in jury trials because they want to appear impartial. Most reports of crimes are filed with U.S. law enforcement agencies, which are responsible for investigating crimes. As long as a statute of limitations has not run, no time limitation exists in the U.S. for criminal investigations. In the U.S., prosecutors decide whether to indict a suspect and for what crimes. The judiciary does not interfere with prosecutors’ discretion to determine the criminal charges against a defendant unless there is a violation of a defendant’s constitutional rights. In the U.S., judges do not supervise criminal investigations. Like in Italy, U.S. defendants have the right to remain silent.

The U.S. judiciary is also independent. The U.S. has two types of judges: state or federal. Most state judges are elected while federal judges are appointed for life. As in Italy, most states and the federal courts use a three tier system of courts: trial courts, intermediate appellate courts, and a supreme court. In the U.S. defendants for serious offenses (i.e., usually crimes that are punishable by more than 6 months in prison), have the right to a jury trial, and the jury’s verdict must be unanimous. U.S. appellate courts generally including the highest court in a jurisdiction can review cases on both factual and legal grounds. The U.S. Supreme Court and the highest state court generally, however, only hear cases when there is an important legal issue in dispute. In the U.S., jurors play no role in appeals.

## Materials and Methods

### Participants

The questionnaire was distributed by E-mail (with link to the web questionnaire) to members of the organization *Laboratorio Esame e Controesame* (LAPEC). Respondents (*n* = 100), six of whom also had acted as prosecutors, were geographically distributed all over Italy. There were 60 males and 40 females; their mean age was 43.7, and they had been attorneys for an average of 14.4 years.

### Questionnaire

The questionnaire was an Italian translation of the Wise and Safer questionnaire (Wise and Safer, 2004; Magnussen et al., 2008). To increase the response rate, the questionnaire covered fewer eyewitness factors than some other surveys (e.g., Desmarais and Read, 2011; Kask, 2011). Eyewitness factors were selected for the questionnaire when there was strong agreement among experts (Kassin et al., 2001) and good empirical support for how the factor affects accuracy. In addition the questionnaire covered some eyewitness factors not included in other surveys, as detailed below.

The participants were asked (a) to respond to 12 eyewitness statements and (b) provide the personal background information summarized in the preceding paragraph. The statements probed respondents’ beliefs and knowledge about eyewitness factors that affect accuracy and are presented in Table 1. The respondents indicated whether they agreed or disagreed with a statement (1–6, 12) or whether they believed the statement was generally true or generally false (statements 7–11). They could also have responded to the statements that they “don’t know” or were “uncertain” of the answer. We determined the correct answer to the statements based on the responses of eyewitness experts (Kassin et al., 2001), and our prior reviews of the eyewitness literature (Wise and Safer, 2004; Magnussen et al., 2008; Wise et al., 2009).

**Table 1:** **Eyewitness topics and statements and the percentage correct (rounded) for various legal groups**.

Topic	Statement	Percent correct				
		Italian defense attorneys (%)	U.S. defense attorneys (%)	U.S. prosecutors (%)	U.S. judges (%)	Norwegian judges (%)
Effects of a hat (A)	It is significantly harder for a witness of a crime to recognize a perpetrator who is wearing a hat during the commission of a crime than a perpetrator who is not wearing a hat	66	56	41	44	55
Minor details (D)	A witness’s ability to recall minor details about a crime is a good indicator of the accuracy of the witness’s identification of the perpetrator of the crime	20	53	7	23	31
Attitudes and expectations (A)	An eyewitness’s perception and memory for an event may be affected by his or her attitudes and expectations	98	99	88	94	98
Conducting lineups (A)	A police officer who knows which member of the lineup or photo array is the suspect should not conduct the lineup or photo array	94	95	42	62	84
Effects of post-event information (A)	Eyewitness testimony about an event often reflects not only what a witness actually saw but information obtained later on from other witnesses, the police, the media, etc.	91	96	66	84	94
Confidence-accuracy (D)	At trial, an eyewitness’s confidence is a good predictor of his or her accuracy in identifying the defendant as the perpetrator of the crime	34	82	22	33	31
Confidence malleability (T)	An eyewitness’s confidence can be influenced by factors that are unrelated to identification accuracy	85	98	82	89	85
Weapon focus (T)	The presence of a weapon can impair an eyewitness’s ability to accurately identify the perpetrator’s face	73	88	50	69	68
Mug-shot-induced bias (T)	Exposure to mug shots of a suspect increases the likelihood that the witness will later choose that suspect from a lineup	93	95	71	74	84
Lineup presentation format (T)	Witnesses are more likely to misidentify someone in a culprit-absent lineup when it is presented in a simultaneous (i.e., all members of a lineup are present at the same time) as opposed to a sequential procedure (i.e., all members of a lineup are presented individually)	48	59	22	19	38
Forgetting curve (T)	The rate of memory loss for an event is greatest right after the event and then levels off over time	87	34	21	31	51
Jurors distinguish eyewitnesses (D)	Jurors can distinguish between accurate and inaccurate eyewitnesses	56	89	38	39	40

*(A) Indicates agreement with the statement is scored as correct*.

*(D) Indicates disagreement with the statement is scored as correct*.

*(T) Indicates a response of “generally true” is scored as correct*.

Respondents also answered questions about whether attorneys, judges, and jurors were knowledgeable about the eyewitness factors, whether defendants should be convicted solely on the basis of eyewitness testimony only in exceptional circumstances, and how frequently eyewitness error is involved in wrongful convictions. Finally they were asked about their exposure to educational materials about eyewitness testimony.

## Results and Discussion

Results of the eyewitness questionnaire are shown in Table 1. For some eyewitness statements (3, 5, 7, 9), a large percentage of the Italian attorneys gave the correct answer, which was also true of the U.S. attorneys (Wise et al., 2009), U.S. and Norwegian judges (Wise and Safer, 2004; Magnussen et al., 2008), and Norwegian lay persons with jury experience (Magnussen et al., 2010). For most statements, however, the percentage of correct answers for the Italian sample is quite modest, as was true of other surveys of legal professionals. In general, however, the responses of the Italian defense attorneys are more similar to the responses of the U.S. defense attorneys than to the responses of the US prosecutors and US and Norwegian judges. The Italian defense attorneys’ responses in general, however, were somewhat less accurate than the responses of the U.S. defense attorneys. The overall score of the Italian defense attorneys was 71% correct, which is somewhat lower than the score of the U.S. defense attorneys (79%), but higher than the scores of U.S. prosecutors (46%) U.S. judges (57%), Norwegian judges (66%), and Norwegian lay persons with jury experience (53%) to the same questions in previous surveys (Wise and Safer, 2004; Magnussen et al., 2008, 2010; Wise et al., 2009). Interestingly, the U.S. prosecutors were the least knowledgeable legal professionals in the surveys, even scoring below the Norwegian jurors.

For statement 11, the Italian defense attorneys gave more correct answers than the other legal professionals in any of the other surveys. This result may have occurred because statement 11 may have been less clear in English than in Italian (Desmarais and Read, 2011).

We also asked the Italian defense attorneys how knowledgeable other legal professions are about eyewitness testimony. A total of 73% of the Italian defense attorneys agreed that attorneys are knowledgeable about eyewitness testimony, but only 51% of them agreed that judges are knowledge. In short, the Italian defense attorneys believed that attorneys know more about eyewitness testimony than judges. Surveys of legal professionals about eyewitness testimony have consistently found that groups believe their members know more about eyewitness evidence than other types of legal professionals (Wise and Safer, 2010).

### Perceptions of eyewitness reliability and its role in wrongful convictions

To assess the attorneys’ views of the reliability of eyewitness testimony, we asked them whether they agreed or disagreed with the following statement: “only in exceptional circumstances should a defendant be convicted of a crime solely on the basis of eyewitness testimony.” Of the Italian defense attorneys, 59% agreed with this statement. In the US samples, 70% of the defense attorneys agreed with it, whereas 61% of the prosecutors disagreed with this statement. A total of 23% of the U.S. judges and 36% of the Norwegian judges agreed with this statement, and 42% of the Norwegian juror sample also agreed with it. Thus the responses of the Italian defense attorneys were more similar to the US defense attorneys than to the other legal professionals, who were much less skeptical regarding jurors’ knowledge of eyewitness testimony and the accuracy of eyewitness testimony.

The attorneys were also asked to indicate out of 100 cases of wrongful felony convictions, how many they thought on average would be due at least in part to eyewitness error. A conservative estimate is that eyewitness error occurs in at least half of all wrongful felony convictions in the U.S. (Scheck et al., 2000). The mean estimate for the Italian defense attorneys was 35 cases, which is similar to the US prosecutors. The mean estimate of the US defense attorneys was 60 cases (Wise et al., 2009). Thus, for this question the estimate of the Italian sample was more similar to the US prosecutors’ estimate than to the defense attorneys’ estimate.

### Exposure to educational material

What has been the extent of the exposure of legal professional to educational materials about eyewitness testimony? Of the Italian defense attorneys, 30% reported reading an article or book or attending a seminar on eyewitness testimony, 27% reported exposure to two of these sources, and 21% had exposure to all three sources, whereas 17% reported no exposure to educational material. In the US samples, a significantly lower percentage of prosecutors than defense attorneys reported reading a law review or psychological article about eyewitness testimony, attending a lecture or seminar on eyewitness testimony, or reading a book on eyewitness testimony. In addition 15% of US prosecutors and 5% of the US defense attorneys answered that they had no exposure to educational materials about eyewitness testimony. The corresponding value for Norwegian judges was 19%. Moreover, 75% of the U.S. prosecutors and 94% of the US defense attorneys, and 95% of the Italian defense attorneys believed that attorneys need more eyewitness training. Ironically, the samples with greater knowledge about eyewitness testimony were the groups who believed they needed more training. Conversely, individuals who were relatively uninformed about eyewitness testimony were more likely to underestimate how much there is to know about it (Wise and Safer, 2010).

### Correlates of attorneys’ knowledge of eyewitness testimony

The 12 eyewitness statements in the questionnaire were combined into a knowledge scale, which measured the number of correct responses the Italian defense attorneys gave to the eyewitness statements. The knowledge scale, correlated with two variables: respondents’ belief that defendants should be convicted of a crime solely on the basis of eyewitness testimony only in exceptional circumstances and with their estimates of the percentage of wrongful convictions that involve eyewitness error (see Tables 1 and 2). As indicated by Table 2, these correlations were low to moderate, suggesting that knowledge of eyewitness testimony may be modestly related to greater skepticism about the value of that testimony. The sizes of the correlations of these two variables with the knowledge scale were similar for the Italian defense attorneys and the U.S. defense attorneys and prosecutors (Wise et al., 2009).

**Table 2:** **Correlations of the knowledge scale with other variables for various legal groups**.

Topic	Italian defense attorneys	U.S. defense attorneys	U.S. prosecutors	U.S. judges	Norwegian judges
Solely from eyewitness testimony	−0.30**	−0.21**	−0.21	−0.35**	−0.20*
Percentage of wrongful convictions	0.32**	0.18**	0.27*	0.21*	0.16
Years as attorney	0.18	0.15**	−0.07	−0.01	−0.01

*These correlations are based upon the 12-item knowledge scale in Table 1, which is slightly different than the knowledge scale used in previous publications involving the U.S. and Norwegian samples*.

*p < 0.05; **p < 0.01.

For the Italian defense attorneys, a greater number of correct responses on the knowledge scale were related to beliefs and attitudes that may be necessary for attorneys to reduce eyewitness error. Moreover, there was little or no relationship between the number of correct answers to the statements and legal experience (see Table 2). For example, the number of years an Italian defense attorney practiced law was not related to knowledge. Thus, the data offers some support for the hypothesis that knowledge about eyewitness factors may be related to attitudes about eyewitness testimony rather than to legal experience. It is also possible that more knowledgeable legal professionals tend to have attitudes and beliefs that are necessary to reduce eyewitness error. This result is similar to what was found in the U.S. and Norwegian surveys of judges (Wise and Safer, 2004; Magnussen et al., 2008). Interestingly, however, the majority of both prosecutors and defense attorneys who participated in the surveys believed that attorneys would benefit from additional training on eyewitness testimony, and also appeared to believe that eyewitness knowledge is not just common sense (Benton et al., 2006).

## General Discussion

The results of the present study confirm the results of the Wise et al. (2009) survey that defense attorneys generally give more correct answers to questions about memory and eyewitness factors than other legal professionals. Comparing the results for the Italian defense attorneys to the results for U.S. prosecutors and defense attorneys and the U.S. and Norwegian judges, shows that the Italian defense attorneys scored closer to the U.S. defense attorneys than to the other samples of U.S. and European legal professionals. The Italian defense attorneys’ responses in general, however, were less accurate than the responses of the U.S. defense attorneys. On every single item, however, the Italian defense attorneys scored higher than the US prosecutors and closer to the U.S. defense attorneys; they also scored higher than U.S. and Norwegian trial judges on every item. Thus, it is unlikely that the superior performance of the U.S. defense attorneys (Wise et al., 2009) is a chance finding. Furthermore, the Italian defense attorneys scored higher on almost every statement than the Norwegian judges who outperformed the U.S. judges (Magnussen et al., 2008). Accordingly, the differences between the U.S. and European criminal justice systems and how the participants for the surveys were recruited also cannot explain the results. Rather it appears that the role of the defense attorneys in the criminal justice system explains their superior performance in the surveys, which raises an additional question. Was the defense attorneys’ superior performance on the survey a product of their greater knowledge of eyewitness testimony or their skepticism of eyewitness testimony?

Wise et al. (2009) found evidence for both hypotheses. The present results, however, show no correlation between the Italian defense attorneys’ scores on the knowledge scale and their exposure to educational materials about eyewitness testimony. Furthermore, the Italian defense attorneys did not have greater exposure to educational materials about eyewitness testimony than did the U.S. prosecutors. In short, it appears that the Italian defense attorneys’ superior performance in the survey was due, to some extent, to their skepticism about the accuracy of eyewitness testimony rather than because of their greater knowledge. Consequently, they likely lack sufficient knowledge to identify the circumstances when eyewitness error is likely to occur, which would hamper their ability to effectively defend their clients from erroneous eyewitness testimony.

The Italian defense attorneys gave a lower estimate of the role of eyewitness error in wrongful felony convictions than the U.S. defense attorneys. According to Wise et al. (2009), a conservative estimate is that eyewitness error occurs in at least half of all wrongful felony convictions in the United States. The estimate of the U.S. defense attorneys was close to 60%, whereas the U.S. prosecutors’ estimate was approximately 35 cases out of 100. The mean estimate for the Italian defense attorneys was almost identical to the U.S. prosecutors. This difference may reflect cultural differences and differences in the European and U.S. criminal justice systems. In the U.S. DNA exoneration cases, eyewitness error occurred in more than 75% of the cases (see text footnote 1; Saks and Koehler, 2005). Analysis of the causes of wrongful convictions in Norway, however, shows that eyewitness error is not a major factor even for comparable cases to the U.S. DNA exoneration cases though the number of wrongful convictions identified in Norway is small (Stridbeck and Magnussen, 2012). Reliable cross-national statistics on the role of eyewitness errors in wrongful convictions would be illuminating. It may be that U.S. defense attorneys, who are more familiar with the DNA exoneration cases, are more sensitive to eyewitness error than their European counterparts and thus more motivated to learn about the cause of eyewitness error.

There are many differences between U.S. and European criminal justice systems and how attorneys and judges are trained and selected, which may impact their views of the accuracy of eyewitness testimony (Stridbeck and Granhag, 2010). Nonetheless, the results of the present study suggest that the special role of the defense attorneys in criminal justice systems makes them more skeptical of eyewitness accuracy than other legal professionals.

In conclusion, the various cross-national surveys have consistently found that legal professionals lack knowledge about eyewitness evidence, but that they generally desire additional education on the topic. New research should develop methods of training new legal professionals, as well as retraining current professionals, to better understand how memory works, the factors that affect eyewitness accuracy, and the legal safeguards necessary to minimize eyewitness error.

## Conflict of Interest Statement

The authors declare that the research was conducted in the absence of any commercial or financial relationships that could be construed as a potential conflict of interest.

## Footnotes

^1^www.innocenceproject.com
